# Creation of EGD-Derived Gastric Cancer Organoids to Predict Treatment Responses

**DOI:** 10.3390/cancers15113036

**Published:** 2023-06-02

**Authors:** Hannah G. McDonald, Megan M. Harper, Kristen Hill, Anqi Gao, Angelica L. Solomon, Charles J. Bailey, Miranda Lin, Mautin Barry-Hundeyin, Michael J. Cavnar, Samuel H. Mardini, Prakash J. Pandalai, Reema A. Patel, Jill M. Kolesar, Justin A. Rueckert, Lawrence Hookey, Mark Ropeleski, Shaila J. Merchant, Joseph Kim, Mei Gao

**Affiliations:** 1 Division of Surgical Oncology, Department of General Surgery, University of Kentucky, Lexington, KY 40536, USA; hmc236@uky.edu (H.G.M.); mei.gao@uky.edu (M.G.); 2 College of Pharmacy, University of Kentucky, Lexington, KY 40536, USA; 3 Division of Gastroenterology, Department of Internal Medicine, University of Kentucky, Lexington, KY 40536, USA; 4 Division of Medical Oncology, Department of Internal Medicine, University of Kentucky, Lexington, KY 40536, USA; 5 Department of Pathology and Laboratory Medicine, University of Kentucky, Lexington, KY 40536, USA; 6 Division of Gastroenterology, Department of Internal Medicine, Queen’s University, Kingston, ON K7L 3N6, Canada; 7 Division of General Surgery and Surgical Oncology, Queen’s University, Kingston, ON K7L 3N6, Canada

**Keywords:** gastric cancer, chemoresistance, organoids

## Abstract

**Simple Summary:**

Gastric cancer is a deadly disease with no established method to choose the most effective chemotherapy for each patient. To address this public health issue, our group has developed a novel approach using patient tumor samples to create 3D tumor models for rapid drug sensitivity testing. We demonstrated the ability to use patient tumor samples that have been shipped overnight to show that selecting the most effective chemotherapy regimen can be accomplished for patients across the nation. We were able to create 3D tumor models within 24 h and perform drug sensitivity testing within 2 weeks of receiving the tumor sample. This indicates that we have developed a methodology to select the most effective chemotherapy for each patient with gastric cancer within two weeks of diagnosis.

**Abstract: Background:**

Gastric adenocarcinoma (GAd) is the third leading cause of cancer-related deaths worldwide. Most patients require perioperative chemotherapy, yet methods to accurately predict responses to therapy are lacking. Thus, patients may be unnecessarily exposed to considerable toxicities. Here, we present a novel methodology using patient-derived organoids (PDOs) that rapidly and accurately predicts the chemotherapy efficacy for GAd patients. **Methods:** Endoscopic GAd biopsies were obtained from 19 patients, shipped overnight, and PDOs were developed within 24 h. Drug sensitivity testing was performed on PDO single-cells with current standard-of-care systemic GAd regimens and cell viability was measured. Whole exome sequencing was used to confirm the consistency of tumor-related gene mutations and copy number alterations between primary tumors, PDOs, and PDO single-cells. **Results:** Overall, 15 of 19 biopsies (79%) were appropriate for PDO creation and single-cell expansion within 24 h of specimen collection and overnight shipment. With our PDO single-cell technique, PDOs (53%) were successfully developed. Subsequently, two PDO lines were subjected to drug sensitivity testing within 12 days from initial biopsy procurement. Drug sensitivity assays revealed unique treatment response profiles for combination drug regimens in both of the two unique PDOs, which corresponded with the clinical response. **Conclusions:** The successful creation of PDOs within 24 h of endoscopic biopsy and rapid drug testing within 2 weeks demonstrate the feasibility of our novel approach for future applications in clinical decision making. This proof of concept sets the foundation for future clinical trials using PDOs to predict clinical responses to GAd therapies.

## 1. Introduction

Gastric adenocarcinoma (GAd) is the third leading cause of cancer-related death worldwide [1,2]. Surgical resection remains the only curative option for patients with early-stage disease [1]. However, for the nearly 40% of patients with locally advanced disease, surgery alone results in high rates of recurrence [1]. Therefore, a multidisciplinary therapeutic approach with neoadjuvant and adjuvant regimens has been applied for locally advanced GAd [1,2,3,4,5]. Current systemic options include FLOT (5-Fluorouracil, Leucovorin, Oxaliplatin, and doceTaxel), ECF (Epirubicin, Cisplatin, and 5-Fluorouracil), FOLFIRI (FOLinic acid, 5-Fluorouracil, and IRInotecan), or FOLFOX (FOLinic acid, 5-Fluorouracil, and OXaliplatin), all of which have shown clinical efficacy in GAd [6,7,8]. In current practice, most patients are administered these regimens without having the benefit of biomarkers to accurately predict treatment responses, as are available in other histological material such as pancreatic and colorectal adenocarcinoma [8,9]. To increase the likelihood of treatment efficacy and to avoid unnecessary toxicities, a precise and expeditious drug-screening model to select the regimen most likely to work for each individual patient is warranted. 

Models to predict treatment response are needed to tailor therapy. Data have shown that patient-derived xenografts (PDXs) may accurately predict treatment responses to better guide the selection of individualized drug options [10,11]. Unfortunately, the time required to establish and test such personalized tumor models is impractical for clinical applications [12]. Therefore, an accurate pre-clinical model that can be created and tested within the period of time needed for clinical decision making is needed in GAd [7,11,13]. Here, we examined patient-derived organoids (PDOs) which have become attractive models for studying tumor biology, developing novel biomarkers, and screening drugs [7,10,11,13,14,15,16,17,18,19,20]. Our group has previously developed PDOs from endoscopic biopsy tissues in patients undergoing esophagogastroduodenoscopy (EGD) [21]. We observed that these PDOs preserved gastric epithelial origin and genomic signatures [21]. Therefore, we theorized that EGD-derived PDOs could be an ideal model for personalized drug sensitivity testing. Recently, we optimized our methodology for drug sensitivity testing using PDO single cells to increase organoid yield and thus, the accuracy of the assay [22]. In this current study, we collaborated with an international medical center to recruit GAd patients and obtain endoscopic biopsy tissues with the objective of evaluating and testing EGD-derived GAd PDOs for personalized therapy applications. 

## 2. Materials and Methods

### 2.1. Patient Recruitment

A collaborative research agreement was formalized with Kingston Health Sciences Centre (KHSC), Kingston, ON, Canada. Institutional Review Board approvals at the University of Kentucky (UK) and the KHSC were obtained for tissue acquisition and analysis. Informed consent was obtained from all GAd patients undergoing EGD to provide biopsy tissues. 

### 2.2. Specimen Collection and Overnight Shipment

**Figure 1:**
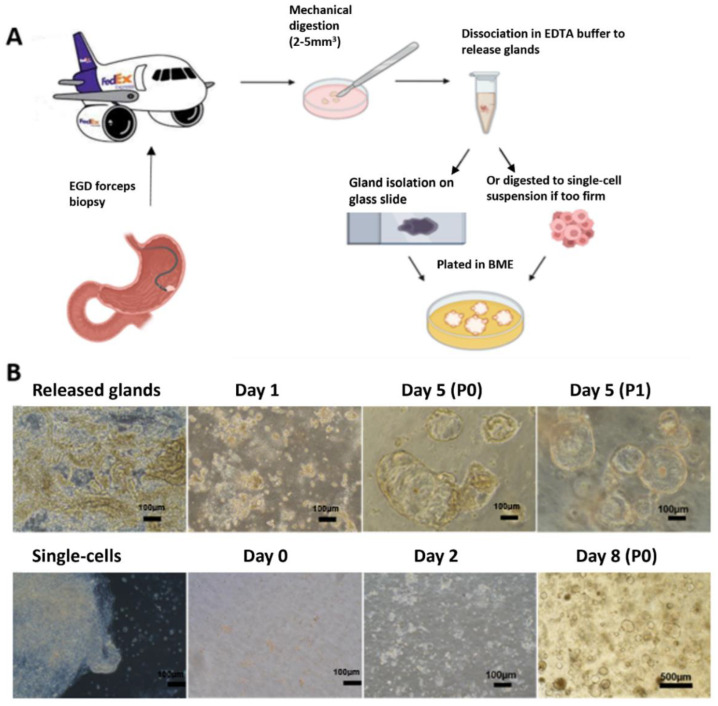
PDO creation from EGD tissues. (**A**) Diagram of the process for PDO creation. A total of 2–3 EGD forceps biopsies were cut into 2–5 mm^3^ pieces in a petri dish and washed thoroughly. Collected pieces were either dissociated with chelating buffer to release glands or they were digested into single cells. Then, the dissociated materials were embedded into BME and overlaid with PDO medium. (**B**) Representative images of PDO creation and growth. Isolated glands from soft tissue grew into relatively large organoids within 3 days (top), while digested single cells from firm tissues formed smaller organoids within 4–7 days (bottom).

Patients (n = 19) underwent EGD at the KHSC or in the UK for initial diagnosis or restaging of GAd. EGD was performed under monitored anesthesia at both institutions. In brief, the endoscope was introduced into the oropharynx and advanced into the stomach. After careful inspection and identification of the suspected malignancy, biopsies using Radial Jaw-4 standard-capacity single-use biopsy forceps (Boston Scientific, Marlborough, MA, USA) were obtained and sent for pathologic evaluation. For research purposes, 2–3 additional forcep biopsies were obtained and suspended in 5 mL low-binding Eppendorf tubes containing PDO wash media advanced DMEM/F12 (AdDF), 1× Penicillin/Streptomycin (Life Technologies, Carlsbad, CA, USA), 1 mL primocin (Invitrogen, Waltham, MA, USA), 10 mM 4-(2-hydroxyethyl)-1-piperazineethanesulfonic acid (HEPES), and 1% Glutamax. The caps of the tubes were tightly sealed with parafilm and wrapped with ice packs in a styrofoam box. For initial testing of the overnight shipment of biopsy tissues, we mailed EGD biopsy specimens from the UK Chandler Hospital to our laboratory research building via overnight FedEx to ensure that the shipped tissues remained in a satisfactory condition. Upon receipt, the biopsy tissues were processed immediately (i.e., within 24 h of initial collection) for PDO development (Figure 1). All subsequent samples from the UK and the KHSC were then shipped overnight to the Kim Laboratory at the University of Kentucky. 

### 2.3. Development of Gastric Cancer PDOs from EGD Specimens

EGD biopsy tissues were separated into two categories, namely soft or firm tissues as described by the gastroenterologist at the time of specimen acquisition (Figure 1). Soft gastric tissues yield a high number of glands. In contrast, firm tissues release few or no glands. Soft biopsy tissues were washed thoroughly and then minced into pieces (2–5 mm^3^) for the isolation of glands with 20 mL of 1× chelating buffer in a 50 mL centrifuge tube for 30 min at 4 °C on a carousel as described [21,22]. Tissue-pieces were then carefully transferred to a 10 cm petri dish and gastric glands were released by pressing the tissues using a glass microscope slide. The glands were then collected with PDO wash media in a 50 mL tube [21,22]. To increase yield, we repeated this dissociation procedure 2–3 times. Released glands were combined, centrifuged at 300 *g* for 5 min, and the resultant pellet was resuspended in 100 µL Cultrex PathClear reduced growth factor basement membrane extract (RGF BME) type 2 (R and D) and plated in 2 wells of pre-warmed 24-well plates at 50 µL/well. BME droplets were allowed to polymerize for 30 min at 37 °C and then overlaid with 500 µL pre-warmed complete organoid medium (28% PDO wash media, 2.5% fetal bovine serum (FBS), 50% conditioned Wnt3A-medium, 20% conditioned R-spondin1 medium, 2% B27, 20 ng/mL human epidermal growth factor; noggin (0.1 μg/mL); 150 ng/mL human fibroblast growth factor-10 (100 ng/mL); 1.25 mM *N*-acetyl-l-cystein; 10 mM nicotinamide; 10 nM human gastrin; and 0.5 μM A83-01). Organoid cultures were kept at 37 °C and 5% CO_2_ in a humidified incubator. They were then maintained at 37 °C and 5% CO_2_ in a humidified incubator as described. Y-27632 (10 μM) was also added to complete organoid media during first seeding and subsequently for passaging [21,22].

For firm biopsy tissues or soft tissues with few or no glands, we used an additional mild digestion technique following the above chelating buffer dissociation to isolate cells from the tissues. Briefly, tissues were rinsed with PDO wash media and then they were minced and incubated in 3 mL digestion media [21,22]. PDO wash media with 0.6 mg/mL collagenase (ThermoFisher, Waltham, MA, USA), 20 mg/mL dispase (ThermoFisher), and 10.5 µM Y-27632 (Tocris, Bristol, UK) for 30 min with gentle agitation [22]. The tubes were then centrifuged at 300× *g* 5 min and the supernatant was removed. The resulting pellet containing dissociated tumor cells was isolated, resuspended in BME, and plated in 2 wells in pre-warmed 24-well plates as mentioned above. 

### 2.4. Maintenance and Analysis of PDOs 

PDOs were maintained and passaged (every 5–7 days) as previously described [21,22]. For passaging, PDOs were either plated in additional wells for expansion or biobanked in PDO media with 10% of FBS and 10% of DMSO and stored in liquid nitrogen for later use. 

When feasible, a small portion of endoscopic biopsy tissues underwent histologic evaluation and analysis prior to PDO creation. In brief, PDOs in BME were plated in a transwell insert (0.4 µm pore, Costar) in 24-well plates at 50–70% confluency and allowed to grow for 1–2 days. The PDO dome was then washed with warm D-PBS and fixed with formalin overnight at room temperature. The PDO dome was then embedded in a drop of prewarmed Histogel (Fisher Scientific, Waltham, MA, USA) on a petri dish. After solidification, the Histogel dome was transferred to a 15 mL falcon tube with 70% ethanol and sent for histologic analysis as described [22]. Hematoxylin and eosin (H and E) staining was performed by the Biospecimen Procurement and Translational Pathology Shared Resource Facility (BPTP SRF) at the University of Kentucky. 

### 2.5. Creation of PDO Single Cells

Following our established protocol, single cells were isolated from standard PDOs at passage 0 (P0) or P1 PDOs as previously described [22]. Single cell PDOs were then plated in pre-warmed 96-well plates at 500–3000 cells/10 µL 50% of BME/well for drug sensitivity testing. 

### 2.6. DNA Extraction and Whole Exome Sequencing

Whole exome sequencing was performed to characterize and compare genomic profiles from the primary gastric cancer and paired standard PDOs and PDO single cells from the same patient. We extracted genomic DNA from primary GAd specimens and paired PDOs and PDO single cells using DNeasy reagents (Qiagen, Hilden, Germany) per the manufacturer’s protocol. We submitted the DNA samples to the Broad Institute for whole exome sequencing (Somatic Exome v6.0). Briefly, an aliquot of genomic DNA (125 ng in 50 μL) was used as the input into DNA fragmentation targeting 385 base pair (BP) fragments. Library preparation was performed using a kit provided by KAPA Biosystems (KAPA Hyper Prep with Library Amplification Primer Mix, product KK8504) and with palindromic forked adapters using unique 8-base index sequences embedded within the adapter (IDT). All steps of library construction and quantification were performed using the Agilent Bravo liquid handling system, while target capture was performed using the Agilent Bravo automated platform. After post-capture enrichment, library pools were quantified using qPCR (automated assay on the Agilent Bravo) using a kit purchased from KAPA Biosystems with probes specific to the adapters. Based on qPCR quantification, pools were normalized using a Hamilton Starlet to 2 nM and sequenced using Illumina Novaseq sequencing technology. All three samples were consistent in copy number alterations and tumor-related gene mutations (Supplemental Figure S1). These results suggest that PDO-derived single cells are representative with regard to the genomics of the primary gastric cancer. 

### 2.7. Drug Sensitivity Testing in PDO Single Cells

Single cells were treated 48–72 h after plating to allow for single cells to reform into uniform miniature PDOs. PDO single cells were treated with the following regimens: ECF, FLOT, FOLFIRI, and FOLFOX. The component ratio in each regimen was based on a fixed set of 5-Fluoruracil (5-FU) concentrations for dose–response curve generation in all four regimens containing 5-FU. Drug components and ratios for each regimen were calculated based on clinical dosages and are listed in Table 1. 5-FU doses were set within a range below C_max_: 0, 0.1, 0.5, 1, 5, 10, 50, and 100 µM and were used as the reference point for the other drugs in each regimen. For drug treatment, culture media was replaced with media containing drug or solvent control (DMSO) and incubated for 48 h. MG132 (10 µM) was used as a positive control. The CellTiter-Glo assay (Promega, Madison, WI, USA) was used to measure cell viability. Plates were analyzed on a Synergy HTX plate reader (BioTek, Winooski, VT, USA). 

### 2.8. Statistical Analyses

Drug testing was performed a single time in 2–3 independent plates or experiments depending on the yield of PDOs for each sample. All data are presented as the mean from 3 replicating wells ± standard deviations for each experiment. The IC_50_ (half maximal inhibitory concentration) and area under the curve (AUC) values were calculated from dose–response curves which were generated with GraphPad Prism 9 software. 

## 3. Results

### 3.1. Patient Sample Collection, PDO Creation

For this study, EGD biopsy tissues from 19 unique GAd patients were shipped overnight from the KHSC (N = 13) or the UK (N = 6). In this cohort, two specimens had no evidence of malignancy and two specimens had contamination. Therefore, a total of 15 GAd EGD biopsy tissues were evaluable (Table 1). Samples included patients with all stages of disease. In the cohort, 13/15 patients received chemotherapy and 13/15 patients received testing for somatic mutations (Table 1). 

Soft tissue specimens developed into PDOs within 1–2 days. Generally, soft tissues had abundant glands and yielded robustly growing PDOs, whereas firm tissues requiring digestion varied in terms of the PDO development and expansion. PDOs were successfully created in 8 out of 15 (53%) patient samples (Table 1). 

### 3.2. PDO Passaging and the Creation of Single Cells 

**Figure 2:**
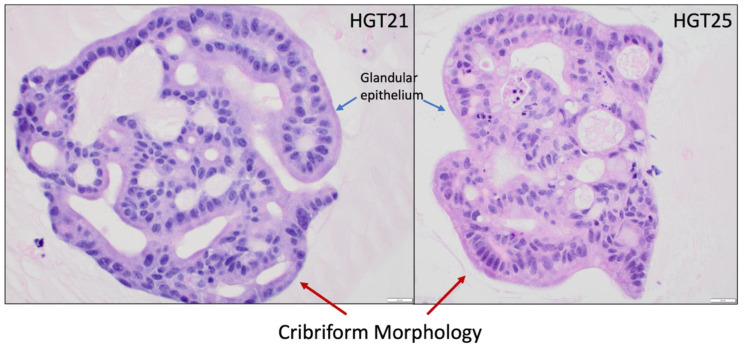
H and E staining of PDOs recapitulate the malignant phenotype. hGT21 is a mixed-type (intestinal/diffuse) GAd while hGT25 is a poorly differentiated adenocarcinoma. All images are in 400× magnification. Scale bar = 20 µm. Blue arrows specify glandular epithelial cells which recapitulate into a cribriform morphology (red arrows) consistent with malignancy.

For PDO creation from limited EGD biopsy tissues, we plated two wells for development (passage 0, P0) and between three and four wells for expansion at P1. For samples with slower-growing PDOs, the original two wells plated at creation (P0) were used for drug-sensitivity testing at P1. Figure 2 shows H and E staining of two EGD-derived PDOs. At day 10–12 after the initial plating, PDOs were dissociated into single cells with Tryple Express enzyme solution as described [22]. We quantified the yield of single cells from two wells of confluent PDOs in the 24-well plate to be 0.5–1 × 10^6^ cells. Single cells were then plated in pre-warmed 96-well plates at 500–3000 cells/10 µL BME/well for drug sensitivity testing. The density of plating depended on the number of single cells obtained from each PDO line. The remainder was used for expansion and banking. 

### 3.3. Combination Drug Testing in Early Passage PDOs

We tested four chemotherapeutic regimens (FLOT, ECF, FOLFIRI, and FOLFOX). In Table 2, the first column lists the component drugs of each regimen. The second column lists the recommended clinical dose of each drug [23]. For each regimen, the components have a fixed dose ratio as shown in the third column. Based on this ratio, we calculated the concentration of each drug for subsequent testing in PDOs by fixing the concentration of 5-FU (0.1, 0.5, 1, 5, 10, 50, and 100 µM) in the combination regimens as shown in the fourth column.

**Figure 3:**
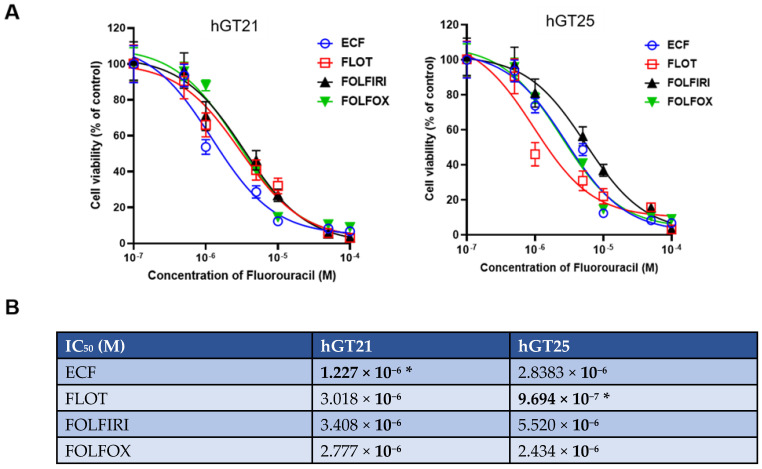
Drug sensitivity testing of combination drug regimens in EGD-derived PDO single cells at passage 2. (**A**) Dose–response curves of hGT21 PDOs showed increased sensitivity to ECF, while hGT25 PDOs showed increased sensitivity to FLOT. (**B**) IC_50_ values of drug regimens in two PDO lines calculated with Graphpad Prism 9. PDO-single cells were plated in 3–6 repeated wells for each treatment. Data are presented as X ± SD. (*) denotes the chemotherapy regimen that the corresponding patient received.

For each combination drug regimen, seven different concentrations of each drug were administered to create dose–response curves for each PDO line [21]. Using this treatment scale, we performed drug sensitivity testing with CellTiter-Glo ATP-based luminescence cell viability assay in P2 PDOs. Cell viability (compared to the control) for each drug concentration was quantified and averaged for three replicates. Dose–response curves were graphed using GraphPad Prism 9 software. IC_50_ and AUC values were then calculated based on the curves. Lower IC_50_ and AUC values indicate higher cytotoxicity and therefore higher efficacy of the drug combination at lower dosages. Figure 3A shows dose–response curves of different drug regimens in PDOs, wherein hGT21 PDOs were most sensitive to ECF and hGT25 PDOs were most sensitive to FLOT. Similarly, Figure 3B shows representative images of the hGT25 PDO single cells treated with the four different regimens, wherein FLOT showed the highest rate of cytotoxicity at the lowest concentrations. When clinical data were available to correlate with drug sensitivity data, the patient associated with hGT21 coincidentally received ECF while the patient associated with hGT25 coincidentally received FLOT. These drug regimens were predicted to provide the highest efficacy. Indeed, both patients have no evidence of disease nearly three years post-gastrectomy.

## 4. Discussion

Current NCCN guidelines support the use of four different chemotherapy regimens, namely FLOT, ECF, FOLFIRI, and FOLFOX, for perioperative management of GAd, yet there is no available method to tailor therapy or predict responses [24]. The development of an assay for the selection of the regimen most likely to work for each individual patient would improve the treatment responses while minimizing unnecessary toxicities. PDOs are an excellent cancer model for such personalized medicine applications due to time efficiency and the preservation of the genomic and histologic makeup of the primary tumor. PDOs for drug sensitivity testing are especially applicable for locally advanced GAd patients whose outcomes are improved with the administration of perioperative chemotherapy. In these patients, the optimal approach for tissue acquisition is endoscopic biopsy. In the pursuit of designing future clinical trials to evaluate PDOs for personalized drug strategies, we previously demonstrated the successful creation of PDOs and PDO single cells from endoscopic biopsies [21,22]. In this study, we demonstrated the rapid development of PDOs for multi-drug sensitivity testing at clinically relevant dosages, even after overnight shipment of biopsy tissues. 

PDXs are the gold standard for patient-derived cancer models [25]. However, PDXs are suboptimal for drug sensitivity testing and clinically actionable decision making. Notably, PDX models can take up to 4–8 months to develop and expand, and consequently, can be quite expensive to maintain [26]. Furthermore, select studies have shown loss of heterozygosity in PDX models over time, raising concerns of altered tumor genomic profiles [12,16]. In contrast, our studies demonstrated the advantages of utilizing PDOs. Firstly, PDOs can be developed from small fractions of tissues (e.g., endoscopic biopsies), especially when large tissue specimens are unavailable or difficult to obtain. Secondly, we demonstrated the feasibility of creating and drug testing PDOs after overnight shipment. Importantly, we confirmed that even when samples originate from distant locations, our methods yielded PDOs that were comparable to those obtained at our home institution. These results provide proof-of-concept that personalized drug testing can be performed for any institution with overnight shipping capabilities. Thirdly, PDOs can be developed and tested within a two-week period, thus avoiding delays in patient care. Finally, the cost of PDO studies is a fraction of what is needed for comparable studies in PDX models. 

PDOs have been shown to recapitulate the tumor microenvironment within the first 14 days of creation, which is critical for in vitro drug testing [27]. Standard drug sensitivity testing protocols generally test monotherapy regimens [15,28,29] or two-drug regimens in one specific case [30]. However, clinical chemotherapy regimens are often multi-drug therapies that cannot be accurately assessed with monotherapy drug testing assays. In this study, we demonstrated the ability to test multi-drug regimens that more closely resemble current clinical applications in an accurate in vitro model. Importantly, in the two patients with drug sensitivity test data, both were treated with the regimen showing the greatest predicted efficacy and both remain without evidence of disease nearly three years post-gastrectomy. 

We identified issues critical to the success of using PDOs for rapid drug sensitivity testing. Firstly, the quality and quantity of endoscopic tissues were essential for success. Since the biopsy tissues can be small, the biopsy forceps must provide adequate tissue sizes (e.g., 5 mm × 5 mm) for the successful creation of PDOs. Additionally, the primary GAd tissues must be characterized as firm or soft to facilitate the appropriate method of dissociation or digestion. With optimization, this methodology shows great promise as a tool to guide treatment for patients with GAd. 

## 5. Conclusions

EGD-derived PDOs can serve as an accurate tool for the prediction of the most effective chemotherapy regimens for individualized care of locally advanced GAd. PDO creation from EGD specimens and subsequent drug sensitivity testing can be achieved within a clinically actionable timeframe even when using overnight-shipped specimens. Furthermore, both patients who received in vitro testing have been disease-free for three years after receiving the regimen with the lowest IC_50_ value. Based on the results of this pilot study, we plan to obtain CLIA certification that will enable future multi-site clinical trials to clinically test whether PDOs can accurately predict the GAd response to therapies. 

## Figures and Tables

**Table 1:** Number of cases listed by histology and the corresponding success rate for generating PDOs. Clinical and pathologic data including stage, chemotherapy administration, and genomic testing results (including HER2 status, PDL1 status, and deficient mismatch repair (dMMR) status) are provided.

Histological Classification	No. of Cases	Success Rate
**Intestinal Type**	**3**	**3/3**
**Diffuse Type**	**4**	**1/4**
**Mixed type (diffuse/intestinal)**	**2**	**2/2**
**Undefined, poorly differentiated**	**3**	**1/2**
**Mucinous adenocarcinoma (poorly differentiated)**	**1**	**1/1**
**Poorly differentiated adenocarcinoma with signet ring cells**	**2**	**0/2**
**Total**	**15**	**8/15 (53%)**
**Stage**	**(n = 15)**	**%**
**I**	**3/15**	**20%**
**II**	**4/15**	**27%**
**III**	**2/15**	**13%**
**IV**	**6/15**	**40%**
**Chemotherapy**	**(n = 15)**	**%**
**Neoadjuvant**	**5/15**	**33%**
**Adjuvant**	**2/15**	**13%**
**Palliative**	**5/15**	**33%**
**Genomic testing**	**Positive/tested**	**%**
**HER2(+)**	**2/13**	**15%**
**PDL1 (+)**	**2/2**	**100%**
**dMMR**	**1/4**	**25%**

**Table 2:** Combination drug regimens and the concentrations for GAd. Four standard of care regimens (FLOT, ECF, FOLFOX, and FOLFIRI) were used. The 5-FU concentration ranged from 50, 10, 5, 1, 0.5, 0.1, to 0 µM in all different regimens with a fixed ratio of other regimen components. The last column is the C_max_ at the single highest clinical dose based on product label of each drug. We observed that the highest tested concentration of each drug was below the maximum exposure plasma concentrations (C_max_).

Drug (Regimens)	Clinical Dosage per Cycle (mg/m^2^)	Fixed Ratio in Combo	Highest In Vitro Testing Concentration (µM)	C_max_ (µM) at Single Highest Clinical Dose
**FLOT**				
**F**luorouracil	**2600**	**52**	**100**	**426**
**L**eucovorin	**200**	**4**	**7.69**	
**O**xaliplatin	**85**	**1.7**	**3.27**	**5**
Doce**T**axel	**50**	**1**	**1.92**	**5.5**
				
**ECF**				
**E**pirubicin	**50**	**1**	**1.2**	**16.6**
**C**isplatin	**60**	**1.2**	**1.42**	**14.4**
**F**luorouracil	**4200**	**84**	**100**	**426**
				
**FOLFOX**				
Leucovorin (**Fol**inic acid)	**200**	**2.35**	**7.12**	
**F**luorouracil	**2800**	**33**	**100**	**426**
**Ox**aliplatin	**85**	**1**	**3.03**	**5**
				
**FOLFIRI**				
Leucovorin (**Fol**inic acid)	**400**	**2.22**	**29.6**	
**F**luorouracil	**3000**	**7.5**	**100**	**426**
**Iri**notecan	**180**	**1**	**13.3**	**5.8**

## Data Availability

Deidentified data are available upon request to the corresponding author. Data are not available on a public repository due to restrictions from our institutional review board.

## Supplementary Materials

The following supporting information can be downloaded at: https://www.mdpi.com/article/10.3390/cancers15113036/s1. Figure S1: whole exome sequencing of tumor, PDO, and single cell.
