# Supplementary material for: Creation of EGD-Derived Gastric Cancer Organoids to Predict Treatment Responses
Source: Cancers (Basel). 2023 Jun 2;15(11):3036. doi: 10.3390/cancers15113036 (PMC10252567; doi:10.3390/cancers15113036)
Supplement: Supplementary file 1 [file cancers-15-03036-s001.zip › cancers-2418480-supplementary.pdf]

A

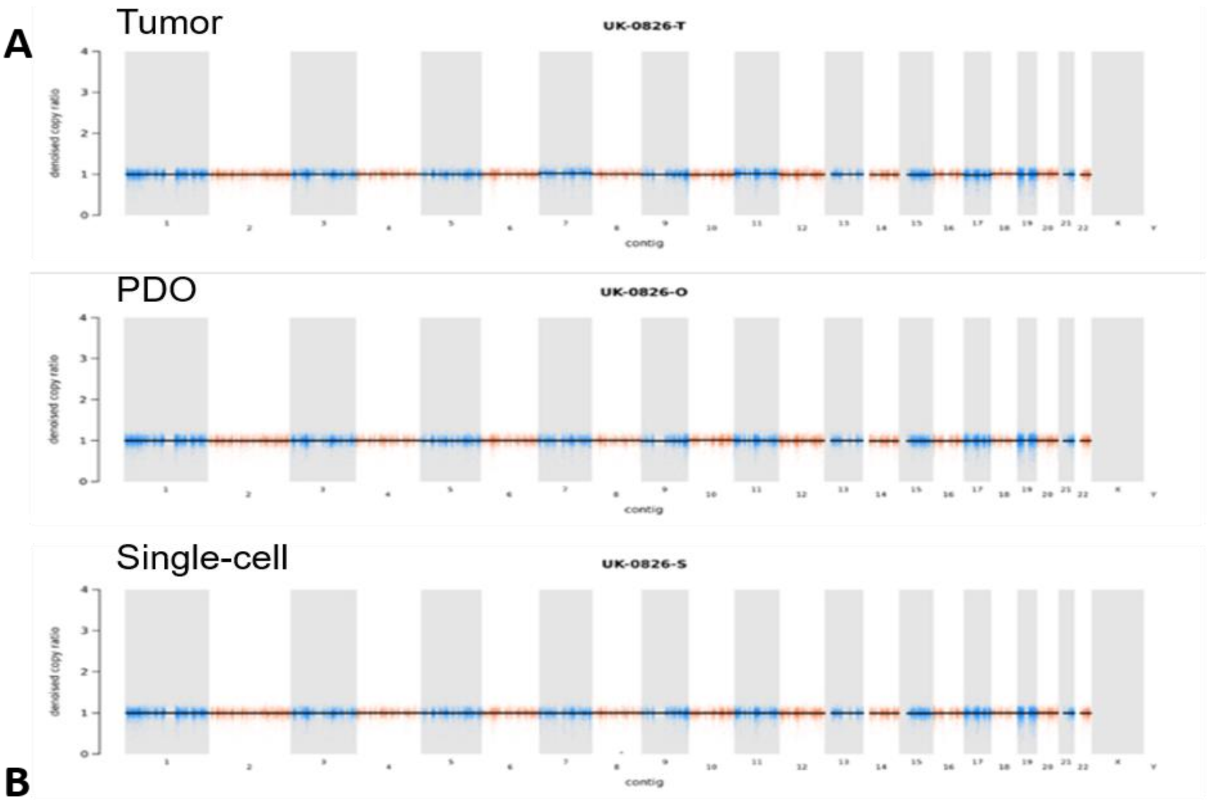

B

Gene	Genome_Change
MSH4	g.chr1:75810760G>A
WDR64	g.chr1:241772878C>T
TMEM247	g.chr2:46480669_46480670insAGCGGCAGCACGAGGTGGTGATGGAGCAGCTGCAGCGGG
MUC12	g.chr7:101002499C>T
CNTNAP3	g.chr9:39132934C>T
CNTNAP3	g.chr9:39133010A>C
ZNF503	g.chr10:75401352_75401354delCCT
FRAT2	g.chr10:97334190_97334227delTAGGGCGCAGCGCGTCCGCGCACGCGGCCGCGGTCCCC
TMEM151A	g.chr11:66295601T>C
KRTAP5-7	g.chr11:71527629_71527630insCTGCTGCCAGTCCAGCTGCTGTAAGCCCTG
GOLGA6L6	g.chr15:20534635_20534718delTCCTCCTGCTCCCGTATCTTCTCCTCCTGCTCCCTTATCTTCTCCTCCTGCTTCCACATCTTCTCCTCCTG[illegible]
MYO5B	g.chr18:49837696_49837697delGC
MYO5B	g.chr18:49837698_49837699insTG
MUC16	g.chr19:8904647_8904648CA>AG
SAMHD1	g.chr20:36898460T>C
SRRD	g.chr22:26483981_26484001delGAGGCGGCGCCCCGGGGGAGA
FAM47A	g.chrX:34130689_34130727delGAGACTGGACGTCCGACGAGTCTTGGGAGGCTCCGAGCG

Figure S1. Whole Exome Sequencing (WES) of primary tumor tissue, standard PDOs, and PDO-derived single-cells from the same GAd patient demonstrates maintenance of genomic integrity in these in vitro models. a) Visualization of copy number variation (CNV) estimated by SNV allelic depth across the genome and depicted in denoised copy ratio plot segment. Individual targets display as points on the plot. Different copy ratio segments are indicated by alternating blue and orange color groups. The denoised median is drawn in thick black. T, primary tumor tissue; O, standard PDOs; S, PDO single-cells. b) Major tumor-related gene alterations (insertion, deletion, and SNP mutation) are similar across the three paired samples from the same patient.
